# Novel Molecular Mechanism of Lenalidomide in Myeloid Malignancies Independent of Deletion of Chromosome 5q

**DOI:** 10.3390/cancers13205084

**Published:** 2021-10-11

**Authors:** Isaac Park, Tra Mi Phan, Jing Fang

**Affiliations:** Department of Drug Discovery and Biomedical Sciences, University of South Carolina College of Pharmacy, Columbia, SC 29208, USA; iipark@email.sc.edu (I.P.); trami@email.sc.edu (T.M.P.)

**Keywords:** immunomodulatory drugs, lenalidomide, myelodysplastic syndromes, cereblon, Ikaros family zinc finger proteins, casein kinase 1 alpha, G protein-coupled receptor 68, regulator of calcineurin 1, calcineurin, cyclosporin

## Abstract

**Simple Summary:**

Lenalidomide is an immunomodulatory drug (IMiD) that has achieved clinical efficacies in multiple myeloma (MM) and myelodysplastic syndromes (MDS) with a single deletion of chromosome 5q (del(5q)). However, many patients treated with lenalidomide relapse and become resistant. Recent studies have demonstrated that lenalidomide binds a protein called cereblon (CRBN), leading to reduced protein levels of IKZF1 and IKZF3 and casein kinase 1 alpha. We have identified signaling molecules downstream of IKZF1, G protein-coupled receptor 68 (GPR68) and regulator of calcineurin 1 (RCAN1) in myeloid malignancies, including MDS and acute myeloid leukemia (AML) with or without del(5q). This review summarizes how lenalidomide exerts anti-tumor activity and highlights novel therapeutic targets that could enhance the anti-tumor activity of lenalidomide with a focus on myeloid malignancies, especially without del(5q).

**Abstract:**

Lenalidomide as well as other immunomodulatory drugs (IMiDs) have achieved clinical efficacies in certain sub-types of hematologic malignancies, such as multiple myeloma, lower-risk myelodysplastic syndromes (MDS) with a single deletion of chromosome 5q (del(5q)) and others. Despite superior clinical response to lenalidomide in hematologic malignancies, relapse and resistance remains a problem in IMiD-based therapy. The last ten years have witnessed the discovery of novel molecular mechanism of IMiD-based anti-tumor therapy. IMiDs bind human cereblon (CRBN), the substrate receptor of the CRL4 E3 ubiquitin ligase complex. Binding of CRBN with IMiDs leads to degradation of the Ikaros family zinc finger proteins 1 and 3 (IKZF1 and IKZF3) and casein kinase 1 alpha. We have found that lenalidomide-mediated degradation of IKZF1 leads to activation of the G protein-coupled receptor 68 (GPR68)/calcium/calpain pro-apoptotic pathway and inhibition of the regulator of calcineurin 1 (RCAN1)/calcineurin pro-survival pathway in MDS and acute myeloid leukemia (AML). Calcineurin inhibitor Cyclosporin-A potentiates the anti-leukemia activity of lenalidomide in MDS/AML with or without del(5q). These findings broaden the therapeutic potential of IMiDs. This review summarizes novel molecular mechanism of lenalidomide in myeloid malignancies, especially without del(5q), in the hope to highlight novel therapeutic targets.

## 1. Introduction

Thalidomide is a derivative of glutamic acid and was banned for its teratogenic effects in pregnant women as a sedative for morning sickness in the 1960s [1]. In 1990s, thalidomide revived due to its anti-inflammation, anti-angiogenesis, anti-tumor and immunomodulatory effects and is approved by the U.S. Food and Drug Administration (FDA) for the treatment of multiple myeloma (MM), a malignancy of plasma cells [2]. Thalidomide and its analogues, lenalidomide and pomalidomide, and newly developed compounds, such as CC-122, CC-220, CC-885 and CC-9009, are known as the immunomodulatory drugs (IMiDs). Lenalidomide and pomalidomide exhibit stronger anti-tumor effect with limited teratogenicity [3,4]. Lenalidomide is used with dexamethasone for patients with MM [5,6,7]. Lenalidomide is also effective in other B cell malignancies, such as chronic lymphoid leukemia (CLL), mantle cell lymphoma (MCL), non-Hodgkin’s lymphoma (NHL), follicular lymphoma (FL) and diffuse large B-cell lymphoma (DLBCL) [8,9,10,11,12,13,14]. Pomalidomide is used for patients with relapsed and refractory (R/R) MM [15]. These are vastly documented and reviewed [16,17,18,19,20,21,22,23,24,25,26] and will not be discussed in this article.

In addition to lymphoid malignancies, lenalidomide is also used for low- or intermediate-1-risk, transfusion-dependent myelodysplastic syndromes (MDS) patients with a single deletion of chromosome 5q (del(5q)) with or without additional cytogenetic lesions [27,28,29]. Accumulating studies have not been able to identify genes on chromosome 5q that exhibit recurrent homozygous inactivation in patients with del(5q) MDS [30]. Instead, heterozygous interstitial deletion of chromosome 5q leads to reduced expression of genes on commonly deleted region (CDR), known as allelic haploinsufficiency, which contributes to clinical phenotype of del(5q) MDS and confers sensitivity to lenalidomide in del(5q) MDS [31,32,33,34,35,36,37,38]. These are discussed and reviewed by many fantastic publications [39,40,41,42,43,44,45,46,47,48,49,50,51,52,53] and will only be very briefly discussed in this article. 

In the last fifteen years, these IMiDs serve as good examples of bench-to-bedside translation to clinical trials and approval by FDA. Since the discovery of the primary target protein of thalidomide, cereblon (CRBN), in 2010 [54], novel molecular mechanism of how these IMiDs execute the anti-tumor activity has been vigorously investigated. These new findings highlight novel therapeutic targets for IMiD-based combination therapy, especially for R/R and more aggressive hematologic malignancies. For example, CRBN acts as the substrate receptor of the CRL4 E3 ubiquitin ligase complex (CRL4^CRBN^) [54,55]. Binding of CRBN with IMiDs alters the recruitment specificity of substrates to the CRL4^CRBN^ complex, leading to their ubiquitination and subsequent degradation by proteasome [55,56]. So far, three CRL4^CRBN^ substrates have been identified that mediate the anti-tumor activity of IMiDs in hematologic malignancies, including the Ikaros family zinc finger proteins 1 and 3 (IKZF1 and IKZF3) [57,58] and casein kinase 1 alpha (CK1α) [59]. CK1α is encoded by a haploinsufficient gene casein kinase 1 alpha 1 (CSNK1A1) that is located on chromosome 5q and is heterozygously deleted in del(5q) myeloid malignancies [32,60]. Haploinsufficiency of CSNK1A1 confers sensitivity to lenalidomide in del(5q) MDS [59]. Of note, we have identified targets of transcription repressor IKZF1 in MDS cells and acute myeloid leukemia (AML) cells, including G protein-coupled receptor 68 (GPR68) and regulator of calcineurin 1 (RCAN1) [61,62,63]. Delineation of the signaling pathways downstream of GPR68 and RCAN1 uncovers novel therapeutic targets that enhance the sensitivity to lenalidomide, especially in R/R and aggressive myeloid malignancies irrespective of del(5q). A good example is Cyclosporin-A that inhibits the activity of calcineurin (CaN), a signaling molecule downstream of RCAN1 [62]. Cyclosporin-A enhances the sensitivity to lenalidomide in MDS/AML with or without del(5q). Cyclosporin-A is an FDA-approved immunosuppressive drug that prevents rejection after organ transplantation [64]. Intriguingly, combined use of lenalidomide and Cyclosporin-A mediates strong tumoricidal effect on MDS/AML cells without damaging T cell function [62].

These discoveries broaden our understanding of the mechanism of action of lenalidomide and provide rationale of novel IMiD-based combination therapy, especially for aggressive sub-type of myeloid malignancies irrespective of del(5q). This review summarizes novel molecular mechanism of lenalidomide with a focus on newly identified CRL4^CRBN^ targets with therapeutic potential and their effect on myeloid malignancies that is independent of del(5q).

## 2. Actions of Lenalidomide 

### 2.1. Anti-Inflammation

As an analogue of thalidomide, lenalidomide is selected due to its potency in inhibiting the production of pro-inflammatory cytokines [65]. In response to lipopolysaccharide (LPS), peripheral blood mononuclear cells (PBMC) produce less pro-inflammatory cytokines, such as tumor necrosis factor alpha (TNF-α), interleukin 1 beta (IL-1β) and interleukin 6 (IL-6), in the presence of lenalidomide [65]. In contrast, treatment with lenalidomide increases the production of anti-inflammatory cytokines, such as interleukin 10 (IL-10) [65].

### 2.2. Immunomodulation

**Figure 1:**
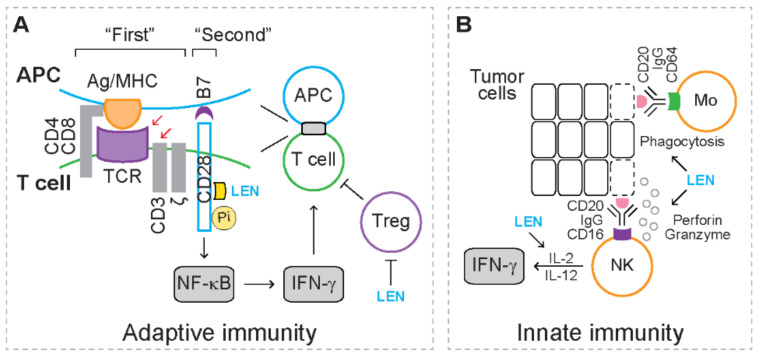
Immunomodulatory effects mediated by lenalidomide. Yellow circles represent phosphorylation (Pi). Yellow square represents lenalidomide (LEN). Dashed rectangles represent dead cells. (**A**) The action of lenalidomide in adaptive immunity. In response to CD3 ligation (red arrow) or antigen-presenting cells (APC, red arrow) (i.e., the “first” signal), lenalidomide phosphorylates co-stimulator receptor CD28 (i.e., the “second” signal), leading to activation of NF-κB, production of interferon gamma (IFN-γ) and proliferation of T cells. Lenalidomide also inhibits proliferation of immunosuppressive regulatory T cells (Treg). Ag/MHC: antigen peptide/major histocompatibility complex; TCR: T cell receptor. (**B**) The action of lenalidomide in innate immunity. When immunoglobulin G (IgG) binds tumor-associated antigens (TAAs), such as CD20, CD16 on natural killer (NK) cells binds IgG, leading to releasing of perforin and granzyme and cytotoxicity of tumor cells. CD64 on monocytes (Mo) binds IgG, leading to phagocytosis of tumor cells. Lenalidomide enhances the antibody-dependent cellular cytotoxicity (ADCC)-mediated by NK cells and monocytes. In the presence of interleukin 2 (IL-2) and interleukin 12 (IL-12), lenalidomide stimulates NK cells to produce IFN-γ.

For adaptive immunity (Figure 1A), upon recognizing the antigen peptide/major histocompatibility complex (MHC) on antigen presenting cells (APCs), T cell receptor (TCR), together with CD3/ζ and coreceptor (i.e., CD4 or CD8) on T cells, activates various downstream signaling pathways [66]. The TCR/CD3/ζ complex and coreceptor serve as the “first” signal to activate T cell response [66]. The co-stimulator receptor CD28 on T cells recognizes the co-stimulator CD80 (also known as B7.1) and CD86 (also known as B7.2) on APCs, serving as the “second” signal to fully activate T cell response [66]. Following CD3 ligation or in response to APCs, lenalidomide induces the production of cytokines, such as interferon gamma (IFN-γ), and promotes the proliferation of human CD4^+^ helper T cells and CD8^+^ cytotoxic T cells [67]. Mechanistic studies reveal that lenalidomide triggers phosphorylation of CD28 on T cells, which leads to activation of NF-κB [67], indicating that lenalidomide acts as a co-stimulator that directly activates the “second” signal during T cell response. In addition, lenalidomide inhibits the proliferation of CD4^+^CD25^+^CTLA-4^+^FoxP3^+^ regulatory T cells (Tregs) that exhibit immunosuppressive activities [68]. These studies suggest that lenalidomide augments T cell response.

For innate immunity (Figure 1B), when immunoglobulin G (IgG) binds tumor-associated antigens (TAAs), the Fc-γ receptors on natural killer (NK) cells (FcγRIII, i.e., CD16) and monocytes (FcγRI, i.e., CD64) recognize the Fc domain on IgG [69,70]. As a result, NK cells mediate tumoricidal effects through releasing perforin and granzymes, while monocytes mediate tumoricidal effect through mediating phagocytosis [69,70]. These tumoricidal effects are known as antibody-dependent cellular cytotoxicity (ADCC). When rituximab (anti-CD20 antibody) recognizes CD20, a lineage marker expressed on lymphoma cells, the addition of lenalidomide potentiates ADCC of NK cells and monocytes [71]. In the presence of IL-2 or interleukin 12 (IL-12), lenalidomide stimulates NK cells to produce IFN-γ when treated with IgG [71]. These studies suggest that lenalidomide enhances the function of NK cells and monocytes.

### 2.3. Anti-Angiogenesis

In an in vitro angiogenesis assay, treatment with lenalidomide reduces sprout formation by human umbilical arterial rings [72]. In a capillary-like cord formation assay, treatment with lenalidomide reduces cord formation by primary endothelial cells [72]. This evidence indicates that lenalidomide exerts anti-angiogenic activity. Lenalidomide-mediated anti-angiogenic activity correlates with disassociation between cadherin 5, beta-catenin and CD31 [72]. Lenalidomide also reduces the expression of hypoxia inducible factor 1 alpha (HIF-1α), a key transcription factor driving angiogenesis [72]. In addition, lenalidomide inhibits growth factor-induced angiogenesis in vivo with a rat mesenteric window assay that is associated with reduced AKT phosphorylation [73]. These studies suggest that lenalidomide exhibits anti-angiogenetic properties.

### 2.4. Anti-Tumor

Accumulating studies reveal that lenalidomide mediates a plethora of direct and indirect anti-tumor effects [63,74]. Lenalidomide induces cytogenetic remission in more than half of the del(5q) MDS patients [27,28,29]. Lenalidomide inhibits cell growth of del(5q) erythroblasts without affecting cytogenetically normal bone marrow cells [31]. Lenalidomide reduces the percentage of myeloid progenitor cells, including CD34^+^CD71^+^ erythroid progenitor cells but increases the proportion of erythroid precursor cells (CD36^+/-^GlycoA^+^) in bone marrow of del(5q) MDS patients, indicating that lenalidomide limits the del(5q) pathological clones and recovers the non-del(5q) clones that exhibit improved erythroid differentiation properties [75]. Lenalidomide also induces more apoptosis in erythroid progenitor and precursor cells from MDS patients than those from healthy controls [75]. In addition to cell-intrinsic effects, lenalidomide also improves the supporting capacity of stromal cells and increases the proportion of activated T cells of MDS patients [75].

The effects of lenalidomide on AML cells are controversial. For example, Wei et al. observe that lenalidomide induces apoptosis accompanied by cell cycle arrest at G2 phase in myeloblasts from a secondary AML patient evolved from MDS with isolated del(5q) [33]. In contrast, no tumoricidal effect is observed in non-del(5q) U937 AML cell line or primary AML cells with normal karyotype in the presence of lenalidomide [33]. Lopez-Millan et al. report no apoptotic effect in del(5q) HL-60 cells, non-del(5q) Molm-13 cells or primary AML cells with normal karyotype or complex karyotype after treatment with lenalidomide [76]. The discrepancy between these two studies is possibly due to different cytogenetic aberrations. Lopez-Millan et al. report that lenalidomide does not overcome the resistance to chemotherapeutic agents in AML cells when they are co-cultured with bone marrow stromal cells [76]. In a different study, Garcia et al. observe that lenalidomide confers vulnerability of myeloblasts from AML patients to chemotherapeutic agents through inducing mitochondrial apoptotic priming [77]. The discrepancy between these two studies is possibly due to whether or not bone marrow stromal cells are involved. Lenalidomide is also shown to induce mobilization of AML cells from bone marrow to peripheral blood, which is associated with reduced expression CXC motif chemokine receptor 4 (CXCR4) [76]. Therefore, more work is needed to clarify the anti-tumor effect of lenalidomide on aggressive sub-types of myeloid malignancies.

## 3. Haploinsufficient 5q Genes Confers Therapeutic Vulnerability to Lenalidomide

**Figure 2:**
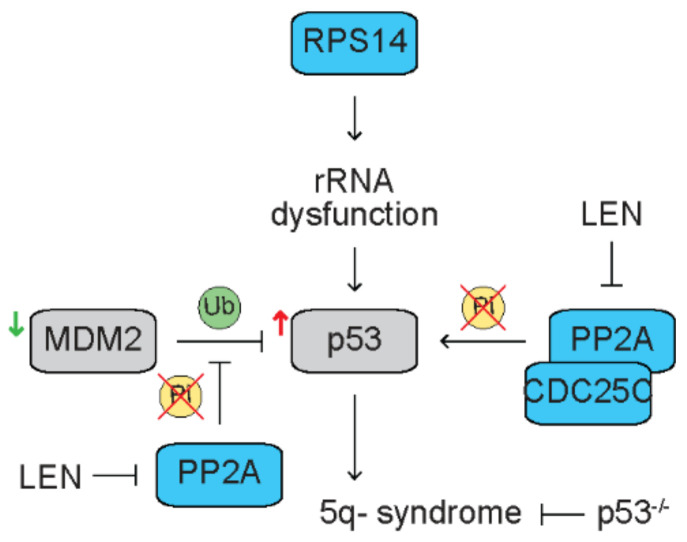
Haploinsufficiency of 5q genes confers therapeutic vulnerability to lenalidomide in del(5q) myeloid malignancies. Blue rectangles represent haploinsufficient 5q genes. Grey rectangles represent diploid genes. Yellow circles represent phosphorylation (Pi). Green circles represent ubiquitination (Ub). Haploinsufficiency of ribosomal protein S14 (RPS14) results in rRNA dysfunction that recapitulates 5q- syndrome through activating p53. Deletion of p53 reverses 5q- syndrome. Mouse double minute 2 (MDM2) ubiquitinates p53 for degradation. In del(5q) MDS patients, p53 is upregulated, while MDM2 is downregulated. Protein phosphatase 2A (PP2A) dephosphorylates p53, abrogates degradation of p53. Lenalidomide (LEN) inhibits PP2A activity, leading to degradation of p53 in AML cells. PP2A dephosphorylates MDM2, dissociating MDM2 with p53 that abrogates degradation of p53. Lenalidomide inhibits PP2A activity, enhancing degradation of p53. Lenalidomide only induces apoptosis and G2 arrest in del(5q) AML cells with haploinsufficiency of PP2A and cell division cycle 25C (CDC25C).

Lenalidomide induces cytogenetic remission in ~50% of del(5q) MDS patients [27,28,29]. Notably, lenalidomide specifically limits the pathological del(5q) clones without damaging the cytogenetically normal clones [31]. This correlates with upregulation of the secreted protein acidic and rich in cysteine gene (SPARC), a haploinsufficient gene localized in the CDR on chromosome 5q [31]. Several more haploinsufficient 5q genes, such as cell division cycle 25C (CDC25C) and protein phosphatase 2A catalytic domain alpha (PP2Acα), are identified that execute the anti-tumor effect of lenalidomide in del(5q) MDS (Figure 2). Casein kinase 1 alpha 1 (CSNK1A1) is also a haploinsufficient 5q gene that is discussed in Section 5.2 [32].

5q- syndrome is a sub-type of MDS with an isolated interstitial deletion of chromosome 5q, a refractory macrocytic anemia and a low risk to progress to AML. The ribosomal protein S14 (RPS14) is a component of the 40S ribosomal sub-unit and required for 18S pre-rRNA processing [78]. With an RNA interference approach to deplete genes localized in CDR on 5q, Ebert et al. discover that partial loss of function of RPS14 in normal hematopoietic progenitor cells phenocopies 5q- syndrome, including reduced terminally differentiated erythroid cells [79]. Mechanistic studies reveal that loss of function of RPS14 disrupts pre-ribosomal RNA processing [79], suggesting that haploinsufficiency of RPS14 and the resultant dysfunctional rRNA metabolism contributes to the pathogenesis of 5q- syndrome. With a large-scale chromosomal engineering approach, Barlow et al. generate a mouse model containing haploinsufficiency of Rps14 that recapitulates 5q- syndrome, including macrocytic anemia and erythroid dysplasia in bone marrow [80]. Haploinsufficiency of Rps14-mediated erythroid phenotype is rescued when p53 is deleted [80]. Further studies reveal that ribosome dysfunction activates p53, leading to accumulatio of p21, a p53 target, and cell cycle arrest in erythroid progenitor cells [81]. Consistently, expression of p53 is upregulated in erythroid precursors from bone marrow of del(5q) MDS patients [35]. Mouse double minute 2 (MDM2) is an E3 ubiquitin–protein ligase that ubiquitinates p53 for degradation. Expression of MDM2 is downregulated in erythroid precursors from bone marrow of del(5q) MDS patients [35].

The dual specificity phosphatases, CDC25C and PP2Acα, are also located in CDR on 5q and haploinsufficient in del(5q) MDS [33]. CDC25C dephosphorylates cyclin-dependent kinase-1 (CDK-1) and drives cell cycle progress, while PP2A dephosphorylates CDC25C to enter mitosis [82]. Therefore, CDC25C and PP2A are coregulators for G2-M checkpoint. Treatment with lenalidomide induces apoptosis accompanied by cell cycle arrest at G2 phase in del(5q) AML cells but not non-del(5q) AML cells [33]. Depletion of CDC25C and PP2Acα with RNA interference resumes sensitivity to lenalidomide in non-del(5q) MDS/AML cells, indicating that haploinsufficiency of 5q genes confers therapeutic vulnerability to lenalidomide in del(5q) myeloid malignancies [33]. Mechanistic studies reveal that treatment with lenalidomide results in retention of phosphor-Tyr15 on CDK-1 and phospho-Ser216 on CDC25C in U937 AML cell line [33]. Further studies reveal that lenalidomide directly inhibits the phosphatase activity of CDC25C but indirectly inhibits the phosphatase activity of PP2A [33]. Simultaneous depletion of CDC25C and PP2Acα potentiates lenalidomide-mediated G2 arrest and apoptosis in AML cells [33]. PP2A dephosphorylates p53, abrogating its ubiquitination and subsequent degradation [34]. As expected, treatment with lenalidomide results in increased degradation of p53 that correlates with increased phosphor-Thr55 and Ser46 on p53 [34]. In addition, PP2A dephosphorylates MDM2 at Ser166 and Ser186, leading to dissociation of MDM2 with p53 that abrogates ubiquitination and degradation of p53 [83]. Treatment with lenalidomide increases MDM2 phosphorylation at Ser166 and Ser186 through inhibiting PP2A activity, leading to increased p53 degradation [35]. These studies suggest that haploinsufficiency of 5q genes confers therapeutic vulnerability to lenalidomide in del(5q) myeloid malignancies.

## 4. CRBN Is Required for IMiD-Mediated Anti-Tumor Effects

### 4.1. CRBN Is the Primary Target Protein of IMiDs

**Figure 3:**
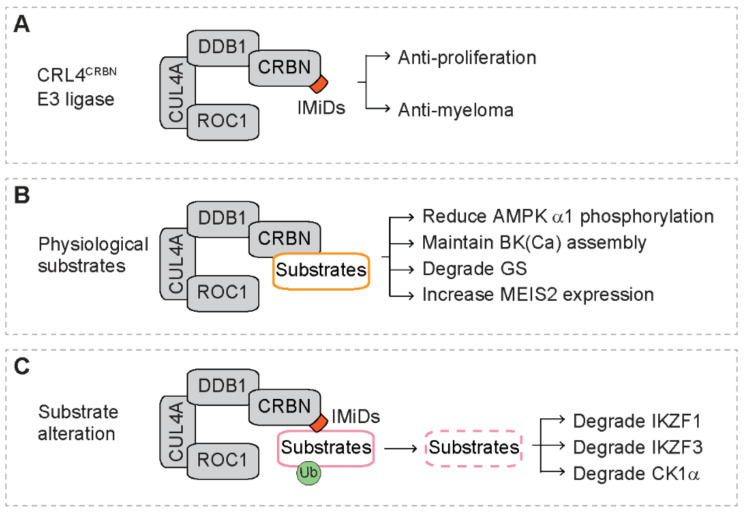
The CRL4^CRBN^ E3 ubiquitin ligase complex. Orange squares represent immunomodulatory drugs (IMiDs). Green circles represent ubiquitination (Ub). Dashed rectangles represent protein degradation. (**A**) General features of the CRL4^CRBN^ complex. The CRL4^CRBN^ complex includes cereblon (CRBN), damaged DNA binding protein 1 (DDB1), cullin 4a (CUL4A) and regulator of cullins 1 (ROC1). IMiDs bind CRBN and exert anti-proliferation and anti-myeloma activity. (**B**) Physiological substrates of CRBN and their consequence after interacting with CRBN. AMPK α1: α1 sub-unit of AMP-activated protein kinase; BK(Ca): large-conductance Ca^2+^-activated K^+^ channels; GS: glutamine synthetase; MEIS2: Meis Homeobox 2. (**C**) Altered substrate recruitment to CRBN in the presence of IMiDs. IKZF1/3: Ikaros family zinc finger proteins 1 and 3; CK1α: casein kinase 1 alpha (CK1α).

With proteomic assays, Ito et al. identify CRBN as the primary target protein that directly binds thalidomide (Figure 3A). With zebrafish and chicken animal models, they also reveal that the interaction of thalidomide with CRBN is required for the teratogenicity [54]. The CRBN gene is involved in mild mental retardation, and the CRBN protein acts as a substrate receptor of the CRL4 E3 ubiquitin ligase complex (CRL4^CRBN^) [54,55]. The CRL4^CRBN^ complex, consisting of CRBN, damaged DNA binding protein 1 (DDB1), cullin 4a (CUL4A) and regulator of cullins 1 (ROC1), catalyzes the ubiquitination of substrates that are delivered to proteasome for degradation [55,56]. Further studies reveal that CRBN also directly binds lenalidomide and pomalidomide, and depletion of CRBN blunts the inhibitory effect of lenalidomide and pomalidomide on proliferation of myeloma cells, indicating that CRBN is required for IMiD-mediated anti-proliferation and anti-myeloma activity [84].

### 4.2. Expression of CRBN Determines Response to Lenalidomide

Several studies reveal that the expression of CRBN is associated with response to thalidomide and lenalidomide in MM patients [85,86,87,88]. Haertle et al. report DNA hypermethylation in an active intronic enhancer within the CRBN locus [89]. Hypermethylation of this region correlates with reduced CRBN expression and reduced response to IMiDs in MM patients [89]. Demethylation of CRBN enhancer confers sensitivity to lenalidomide in MM cells [89]. Lower risk del(5q) MDS exhibit higher levels of CRBN transcripts as compared to lower risk non-del(5q) MDS and healthy control [90]. High levels of CRBN transcripts are observed in MDS patients that respond to lenalidomide [90]. Reduction of CRBN expression correlates with loss of response to lenalidomide and disease progression [90]. These studies suggest that CRBN expression determines response to lenalidomide in MDS and MM.

## 5. Substrates of The CRL4^CRBN^ E3 Ligase Complex

### 5.1. Physiological Substrates

Under physiological conditions, several cellular proteins are found to interact with CRBN (Figure 3B) [91]. For example, CRBN directly binds the α1 sub-unit of AMP-activated protein kinase (AMPK α1) [92]. Overexpression of CRBN reduces phosphorylation of AMPK α1, leading to inactivation of AMPK that is independent of nutrients [92]. CRBN regulates the assembly of the large-conductance, Ca^2+^-activated K^+^ channels (BK(Ca)) in human brains [93]. The mutant CRBN (R419X) disturbs the assembly of BK(Ca) isoform in the adult brain, which is predicted to alter BK(Ca) function and contributes to cognitive impairment in patients with autosomal recessive non-syndromal mental retardation (ARNSMR) mild type [93]. In the presence of high levels of glutamine, glutamine synthetase (GS) is acetylated, followed by binding to the CRL4^CRBN^ complex and subsequent ubiquitination and degradation by the proteasome [94]. With human protein microarrays, Fisher et al. identify several endogenous CRBN substrates, including GRINL1A, MBOAT7, OTUD7B, C6orf141 and Meis Homeobox 2 (MEIS2) [95]. Treatment with IMiDs increases the levels of MEIS2 protein in vitro and in vivo [95].

### 5.2. Substrate Alteration in the Presence of IMiDs

Recent studies demonstrate that instead of recruiting physiological substrates, CRBN recruits a different set of proteins in the presence of IMiDs (Figure 3C) [39]. This leads to ubiquitination and degradation of these proteins, indicating that IMiDs alters the recruitment specificity of substrates to the CRL4^CRBN^ complex [55,56]. The glutarimide ring of IMiDs sits in the substrate binding pocket of CRBN [95,96], suggesting that IMiDs may act to link CRBN and the recruited proteins. Three proteins have been identified to be recruited to the CRL4^CRBN^ complex in the presence of IMiDs, including the Ikaros family zinc finger proteins 1 and 3 (IKZF1 and IKZF3) [57,58] and casein kinase 1 alpha (CK1α) [59]. These newly identified CRL4^CRBN^ substrates mediate the anti-tumor activity of IMiDs in hematologic malignancies.

IKZF1 (also known as Ikaros) and IKZF3 (also known as Aiolos) are transcription factors that function as homodimers and heterodimers. IKZF1 and IKZF3 are essential for terminal differentiation of B cells and T cells [97,98]. IKZF3 is required for the generation of high affinity plasma cells, the physiological counterpart of myeloma cells, during antibody response [99]. Deletion of IKZF1 and IKZF3 is observed in patients with B-progenitor acute lymphoblastic leukemia (ALL) [100], indicating a tumor suppressor function of the Ikaros family. In more mature B cell malignancies, such as CLL, expression of IKZF3 is upregulated, and high levels of IKZF3 correlate with inferior clinical outcome [101], indicating an oncogenic function of the Ikaros family. In addition, IKZF1 and IKZF3 are essential to support the growth and survival of MM cells [57,58]. In 2014, two groups independently reported that, in the presence of lenalidomide, CRBN binds IKZF1 and IKZF3, leading to their ubiquitination and subsequent degradation by proteasome in MM cells [57,58]. Q146 on IKZF1 and Q147 on IKZF3 are required for the binding to CRBN and lenalidomide-mediated degradation [58]. Reduced levels of IKZF1 and IKZF1 are observed in lenalidomide-sensitive but not -resistant myeloma cell lines [57,58]. Depletion of IKZF1 and IKZF3 inhibits the growth of MM cells that are sensitive to lenalidomide [57]. Overexpression of IKZF3 or expression of a IKZF3^Q147H^ mutant that is resistant to lenalidomide-mediated degradation confers resistance to lenalidomide in MM cells [57]. These studies suggest that lenalidomide mediates the anti-myeloma effect through degrading IKZF1 and IKZF3.

**Figure 4:**
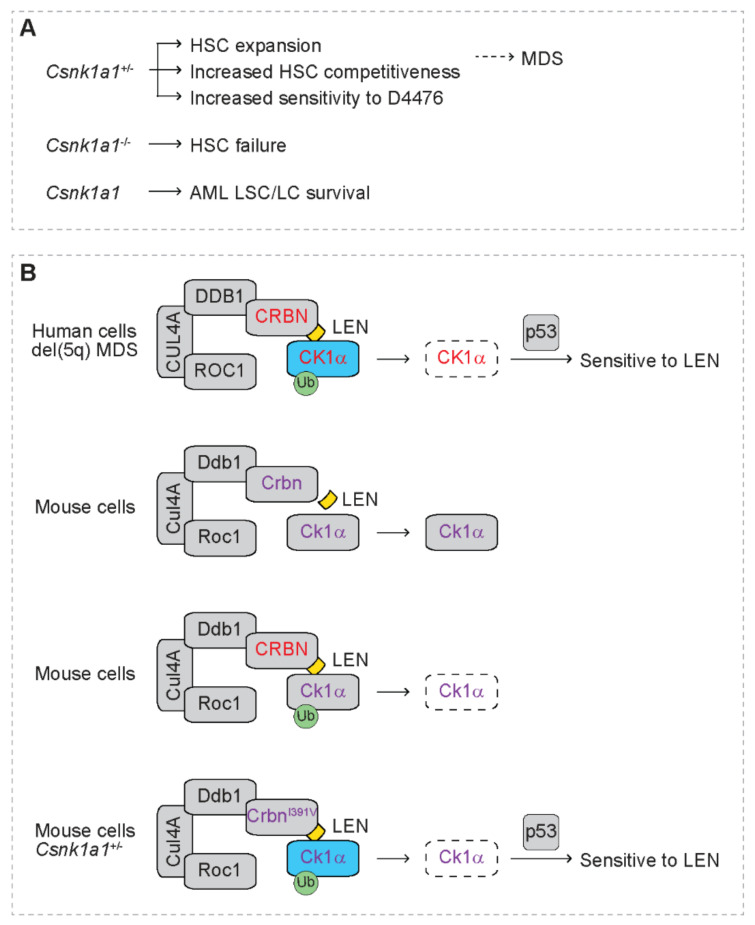
Haploinsufficient *casein kinase 1 alpha 1* (*CSNK1A1*) confers sensitivity to lenalidomide. (**A**) The function of CSNK1A1 in normal and pathological hematopoiesis. Heterozygous deficiency of *Csnk1a1* (*Csnk1a1*^+/−^) in mice leads to hematopoietic stem cell (HSC) expansion and increased competitiveness of HSC, indicating a role of Csnk1a1 in myelodysplastic syndromes (MDS). *Csnk1a1*^+/−^ hematopoietic cells are more sensitive to casein kinase 1 inhibitor D4476. Homozygous deletion of *Csnk1a1* (*Csnk1a1*^−/−^) in mice leads to HSC failure. Csnk1a1 supports the survival of leukemia stem cells (LSC) and leukemia cells (LC). (**B**) Haploinsufficiency of *CSNK1A1* confers sensitivity to lenalidomide. Blue rectangles represent haploinsufficient 5q genes. Grey rectangles represent diploid genes. Dashed rectangles represent protein degradation. Green circles represent ubiquitination (Ub). Yellow squares represent lenalidomide (LEN). Cereblon (CRBN), damaged DNA binding protein 1 (DDB1), cullin 4a (*CUL4A*) and regulator of cullins 1 (ROC1) form the CRL4^CRBN^ E3 ubiquitin ligase complex. In human cells in the presence of lenalidomide, human cereblon (CRBN, red font) binds human casein kinase 1 alpha (CK1α, red font), the protein product of *CSNK1A1*, leading to ubiquitination and degradation of CK1α. Haploinsufficiency of *CSNK1A1* confers sensitivity to lenalidomide in del(5q) MDS through activating p53. In mouse cells in the presence of lenalidomide, mouse Crbn (purple font) does not lead to degradation of mouse Ck1α (purple font). In mouse cells expressing human CRBN (red font), human CRBN leads to degradation of mouse Ck1α (purple font) in the presence of lenalidomide. In mouse cells expressing mutant mouse Crbn^I391V^ (purple font), Crbn^I391V^ leads to degradation of mouse Ck1α (purple font) in the presence of lenalidomide. *Csnk1a1*^+/-^ mouse cells expressing mouse Crbn^I391V^ are sensitive to lenalidomide.

*CSNK1A1* is localized in CDR on 5q and is deleted in del(5q) MDS/AML [32]. Its protein product, casein kinase 1 alpha (CK1α), is a serine/threonine kinase, and its expression level is downregulated in del(5q) MDS cells due to haploinsufficiency of CSNK1A1 [60]. Heterozygous deficiency of Csnk1a1 (Csnk1a1^+/−^) in mice leads to hematopoietic stem cell (HSC) expansion and increased competitiveness [102], indicating that CK1α plays a critical role in the pathogenesis of MDS (Figure 4A). In contrast, homozygous deletion of Csnk1a1 (Csnk1a1^−/−^) in mice leads to HSC failure [102]. Intriguingly, Csnk1a1^+/−^ hematopoietic cells are more sensitive to casein kinase 1 inhibitor D4476 than wild type cells [102]. In addition, CK1α is also required to support the survival of AML cells [103]. Depletion of Csnk1a1 increases p53 activity, leading to selective cytotoxicity in leukemia cells and leukemia stem cells (LSC) but not normal hematopoietic cells [103]. These studies suggest that CK1α is a potential therapeutic target for myeloid malignancies.

Krönke at al. report increased ubiquitination of CK1α that correlates with reduced levels of CK1α protein in AML cell lines after treatment with lenalidomide without altering its transcript levels (Figure 4B) [59]. Lenalidomide treatment also reduces CK1α protein expression in bone marrow cells of AML patients in vivo. Lenalidomide reduces protein expression of both wild type and mutant CK1α. Reduced CK1α protein mediated by lenalidomide is abrogated by proteasome inhibitor MG132 and NAE inhibitor MLN4924, indicating that CK1a is degraded by lenalidomide through proteasome- and cullin-mediated mechanism. In addition, depletion of CRBN reverses lenalidomide-mediated degradation of CK1α. Mechanistic studies reveal that CRBN directly binds CK1α, indicating that CK1α is a direct target of the CRL4^CRBN^ E3 ligase complex in AML cells. Ectopic expression of CK1α but not IKZF1 blunts the sensitivity of del(5q) MDS cells to lenalidomide. Of note, lenalidomide does not decrease CK1α protein in mouse hematopoietic cells, indicating species-specific degradation of CK1α by lenalidomide. Intriguingly, expression of human CRBN resumes lenalidomide-mediated degradation of mouse CK1α. A mutant human CRBN, i.e., CRBN^V387I^, disrupts lenalidomide-mediated degradation of mouse CK1α. Consistently, a mutant mouse Crbn, i.e., Crbn^I391V^, resumes lenalidomide-mediated degradation of CK1α. In addition, Crbn^I391V^ also resumes lenalidomide-mediated degradation of IKZF1 and IKZF3, indicating that substitution of a single amino acid confers response to lenalidomide in degrading substrates. As expected, Csnk1a1^+/−^ hematopoietic cells resume sensitivity to lenalidomide in the presence of Crbn^I391V^, which correlates with induction of p21, the p53 target. Lenalidomide-mediated tumoricidal effect is rescued by heterozygous deletion of p53, indicating that p53 is downstream of CK1α and exerts the tumoricidal effect caused by degradation of CK1α. In contrast, wild type cells are not sensitive to lenalidomide, indicating that haploinsufficiency of CSNK1A1 confers sensitivity to lenalidomide. This is consistent with superior clinical response to lenalidomide in del(5q) MDS patients than those with non-del(5q) myeloid malignancies [27,28,29,104]. This is also consistent with the observation that del(5q) MDS patients with wild type p53 are more sensitive to lenalidomide than those with mutant p53 [105,106].

Intriguingly, treatment with thalidomide, lenalidomide, pomalidomide and CC-122 all result in degradation of IKZF1 [59]. However, only lenalidomide mediates degradation of CK1α. In contrast, thalidomide and CC-122 fail to alter the protein levels of CK1a, while pomalidomide only exhibit weak effects on CK1α protein expression. These suggest that subtle structural difference in IMiDs could impact on substrate specificities. These studies are consistent with the clinical observations that all IMiDs are effective for patients with MM but only lenalidomide exerts hematologic and cytogenetic response in patients with del(5q) MDS [2,5,6,7,15,27,28,29].

## 6. Signaling Pathways Downstream of IKZF1 and IKZF3

**Figure 5:**
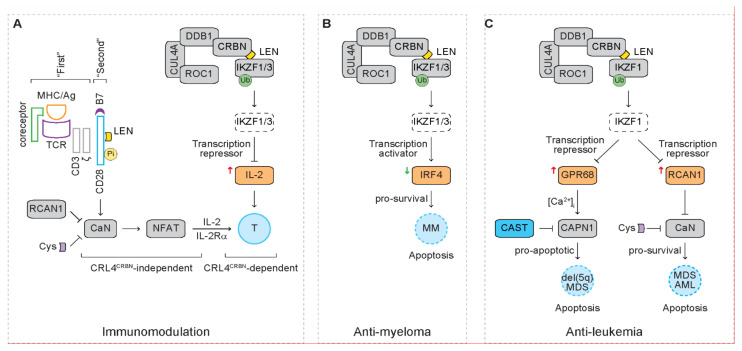
Signaling pathways downstream of IKZF1 and IKZF3 in response to lenalidomide. Blue rectangles represent 5q genes. Grey rectangles represent diploid genes. Dashed rectangles represent protein degradation. Dashed circles represent dead cells. Yellow circles represent phosphorylation (Pi). Green circles represent ubiquitination (Ub). Yellow square represents lenalidomide (LEN). Purple square represents Cyclosporin-A (Cys). (**A**) Lenalidomide executes immunomodulation through CRL4^CRBN^-dependent and -independent mechanism. Cereblon (CRBN), damaged DNA binding protein 1 (DDB1), cullin 4a (CUL4A) and regulator of cullins 1 (ROC1) form the CRL4^CRBN^ E3 ubiquitin ligase complex. T cell receptor (TCR) and coreceptor serve as the “first” signal to activate various signaling pathway, such as calcineurin (CaN)/nuclear factor of activated T cells (NFAT), and finally activate T cells. Lenalidomide could directly phosphorylate CD28, serving as co-stimulator (i.e., “second” signal) that activates T cell response (i.e., CRL4^CRBN^-independent mechanism). Regulator of calcineurin 1 (RCAN1) and Cyclosporin-A (Cys) inhibits the activity of calcineurin and T cell response. In the presence of lenalidomide, the CRL4^CRBN^ complex mediates the degradation of IKZF1/3, leading to increased expression of interleukin 2 (IL-2) that activates T cell response (i.e., CRL4^CRBN^-dependent mechanism). Ag/MHC: antigen and major histocompatibility complex. (**B**) Lenalidomide mediates anti-myeloma effect through depleting the IKZF1/3-IRF4 pro-survival pathway. In the presence of lenalidomide, the CRL4^CRBN^ complex mediates the degradation of IKZF1/3, leading to reduction of interferon regulatory factor 4 (IRF4) and the resultant apoptosis of multiple myeloma (MM) cells. (**C**) Lenalidomide mediates anti-leukemia effect through activating the calpain 1 (CAPN1) pro-apoptotic pathway and/or inhibiting the CaN pro-survival pathway. In the presence of lenalidomide, the CRL4^CRBN^ complex mediates the degradation of IKZF1, leading to increased expression of G protein-coupled receptor 68 (GPR68) that elevates intracellular calcium levels ([Ca^2+^]_i_) and activates calpain 1 pro-apoptotic pathway in del(5q) MDS cells when the physiological calpain 1 inhibitor calpastatin (CAST), a 5q gene, is expressed at lower levels. Meanwhile, degradation of IKZF1 leads to increased expression of RCAN1 that inhibits the CaN pro-survival pathway in MDS/AML. Cyclosporin-A (Cys) enhances the anti-leukemia effect of lenalidomide through inhibiting CaN.

As critical transcription factors for lymphopoiesis and lymphoid malignancies, degradation of IKZF1 and IKZF3 by the CRL4^CRBN^ E3 ubiquitin ligase complex mediates the anti-myeloma effect of lenalidomide [57,58]. Understanding the signaling pathways downstream of IKZF1 and IKZF3 shed light on novel mechanism of action of lenalidomide. In addition to lymphoid malignancies, we have found that IKZF1-mediated signaling pathways also mediate the anti-tumor activity of lenalidomide in myeloid malignancies [61,62,63]. These newly identified signaling pathways provide rationale for future bench-to-bedside translational work (Figure 5). 

### 6.1. IL-2

The Ikaros family transcription factors action as transcription activators or repressors. In mouse Th17 cells, IKZF3 functions as a transcription repressor, silencing the IL-2 locus [107]. Lenalidomide reduces expression of IKZF1 and IKZF3 in human T cells, which correlates with increased IL-2 mRNA expression (Figure 5A) [57]. Depletion of IKZF1 or IKZF3 also correlates with increased IL-2 mRNA expression [57]. As expected, depletion of CRBN abrogates the upregulation of IL-2 mRNA mediated by lenalidomide. These studies suggest that lenalidomide-mediated degradation of IKZF3 derepresses IL-2 transcription in T cells, serving as a mechanism of lenalidomide-mediated immunomodulation that is dependent on the function of the CRL4^CBRN^ complex (i.e., CRL4^CRBN^-dependent).

### 6.2. IRF4

Interferon regulatory factor 4 (IRF4) is a transcription factor required for activation of B cells and differentiation of plasma cells that secrete antibodies during immune response [108,109]. With a loss of function genetic screen, IRF4 is identified to be required for myeloma cell survival through activating its direct target MYC [110]. IRF4 is also a direct target of MYC, generating an auto-regulatory circuit to support myeloma cell survival [110]. Loss of function of IKZF1 and IKZF3 correlates with reduced IRF4 mRNA expression and reduced binding of IKZF1 to IRF4 locus, indicating that IKZF1 and/or IKZF3 act as transcription activators that drive transcription of IRF4 (Figure 5B) [57,58]. These studies suggest that IMiDs mediate anti-myeloma effect through depleting the IKZF1/3-IRF4 pro-survival pathway.

### 6.3. GPR68

To identify the molecular mechanism of the tumoricidal effect of lenalidomide on MDS cells, we have performed a loss of function screen with a genome-wide RNA interference approach in an MDS cell line [61], MDSL, which is derived from a patient with low-risk del(5q) MDS (Figure 5C) [111,112]. In response to lenalidomide, the CD34^+^ compartment of MDSL cells exhibits reduced progenitor cell function as measured by colony-forming cell (CFC) assay. In contrast, the CD34^−^ compartment of MDSL cells exhibits enhanced apoptosis after treatment with lenalidomide. Depletion of CRBN completely reverses the inhibitory clonogenicity of MDSL cells mediated by lenalidomide, indicating that lenalidomide actions through the CRL4^CRBN^ E3 ligase complex in MDSL cells. After treatment with lenalidomide, 24 shRNA clones are identified to be enriched ≥1.5-fold in MDSL cells. Among these enriched shRNA clones, knockdown of G protein-coupled receptor 68 (GPR68), interferon regulatory factor 9 (IRF9), ribosomal RNA processing 1B (RRP1B) or regulator of calcineurin 1 (RCAN1) also reverses the inhibitory clonogenicity of MDSL cells mediated by lenalidomide. In addition, the promoters of GPR68, IRF9 and serum amyloid A1 (SAA1) contain binding peaks for IKZF1. In the presence of lenalidomide, the levels of GPR68 transcript and protein are upregulated in MDSL cells as well as primary MDS cells and normal CD34^+^ hematopoietic stem and progenitor cells (HSPCs). Lenalidomide-mediated upregulation of GPR68 expression is reversed in MDSL cells after expressing a mutant IKZF1 that is resistant to LEN-mediated degradation (i.e., IKZF1^Q146H^) but not wild type IKZF1, indicating that IKZF1 actions as a transcription repressor for GPR68.

GPR68, together with G protein-coupled receptor 4 (GPR4), G protein-coupled receptor 65 (GPR65) and G protein-coupled receptor 132 (GPR132), belongs to the family of proton-sensing G protein-coupled receptor (GPCRs) [113,114,115]. In response to extracellular protons, the histidine residues on the extracellular domains may cause conformational change of the receptors, leading to activation of distinct downstream pathways. For GPR68, Gq/11 is coupled that activates the phospholipase C beta (PLCβ)/calcium (Ca^2+^) pathway when extracellular pH is neutral to mildly acidic (i.e., pH 7.6–6.0) [113]. GPR4, GP65 and GPR68 associate with GS, which activates the adenylyl cyclase (AC)/cyclic AMP (cAMP) pathway [113,116,117]. In addition to interaction with cognate ligands, overexpression of GPCRs is also sufficient to elevate the activity of downstream pathways [118]. Growing evidence implicates that proton-sensing GPCRs regulate the growth and survival of tumor cells, including hematologic malignancies [119]. GPR65 is primarily expressed in immune cells and is downregulated in human myeloid and lymphoid leukemias and lymphomas [120,121,122]. Restoring expression of GPR65 mediates apoptosis of leukemia cells in vitro and retards leukemia expansion in vivo [122], indicating a tumor suppressor function of GPR65 in hematologic malignancies. In contrast, GPR132 is highly enriched in leukemias and lymphomas and is upregulated in primary AML compared to normal hematopoietic cells [123]. The small molecule compound ONC212 that specifically activates GPR132 induces apoptosis in human cell lines of myeloid and lymphoid leukemias and lymphomas [123], indicating a tumor suppressor function of GPR132 in hematologic malignancies. These studies suggest that altered expression of proton-sensing GPCRs could impact on the behavior of hematologic malignant cells.

With a genetic loss of function approach, whole-body Gpr68^−/−^ mice exhibit slightly reduced B cell output with age and upon hematopoietic regeneration [124]. However, no significant alterations on the homeostasis and function of HSC are observed in Gpr68^-/-^ mice either under steady state or upon stress [125]. These studies indicate that GPR68 is dispensable for normal hematopoietic cells and HSC and therefore serves as a potential therapeutic target to enhance the sensitivity to lenalidomide in hematologic malignancies. Further studies reveal that depletion of GPR68 dampens the inhibitory effect of lenalidomide on MDSL cells in vivo [61]. In contrast, the GPR68 agonist N-cyclopropyl-5-(thiophen-2-yl)-isoxaole-3-carboxamide (Isx) enhances the inhibitory effect of lenalidomide on clonogenicity in MDSL cells, indicating that overexpression and/or activation of GPR68 impacts on the response to lenalidomide in MDS cells. Mechanistic studies reveal that lenalidomide-mediated inhibitory effect on clonogenicity and apoptosis in MDSL cells is blunted by EGTA or BAPTA-AM that deplete extracellular and intracellular Ca^2+^ ions, respectively, but facilitated by ionomycin that raises intracellular Ca^2+^ levels, indicating that cytosolic Ca^2+^ levels impact the response to lenalidomide in MDS cells. Short-term treatment with lenalidomide does not induce an instant Ca^2+^ influx in the cytosol of MDSL cells. Intriguingly, long-term treatment with lenalidomide elevates cytosolic Ca^2+^ levels in MDSL cells in vitro and in vivo, which is dependent on the expression of CRBN and GPR68. In addition, lenalidomide induces tumoricidal effects in primary MDS cells as well as three out of seven AML cell lines with or without del(5q) that correlates with elevation of cytosolic Ca^2+^ levels. These data indicate that lenalidomide activates a GPR68/Ca^2+^ pro-apoptotic pathway in MDS/AML cells irrespective of del(5q).

Treatment with lenalidomide enhances the activation of calpains (CAPNs), a family of Ca^2+^-dependent cysteine proteases, that is dependent on the expression of CRBN and GPR68, and CAPN inhibitor PD150606 completely reverses the tumoricidal effect of lenalidomide on MDSL cells and TF-1 cells (an AML cell line with del(5q)), indicating that lenalidomide activates a GPR68/Ca^2+^/CAPN pro-apoptotic pathway in MDS/AML cells. Intriguingly, lenalidomide-sensitive MDS/AML cell lines, including MDSL, KG-1α and F36P, exhibit increased expression of calpain 1 (CAPN1) protein in the presence of lenalidomide that is independent of IKZF1 or GPR68. In contrast, lenalidomide-resistant AML cell lines, including HL-60, THP-1 and Kasumi-1, exhibit comparable CAPN1 protein levels in the presence of lenalidomide. Depletion of CAPN1 reverses the inhibitory effect of lenalidomide on clonogenicity in MDSL cells. These data suggest that CAPN1 is required for executing GPR68/Ca^2+^-mediated cytotoxicity in MDSL cells. To note, the expression of calpastatin (CAST), an endogenous calpain inhibitor that is located on chromosome 5q, is lower in lenalidomide-responders than non-responders, and depletion of CAST increases the sensitivity to lenalidomide in AML cell lines that exhibit cytosolic Ca^2+^ accumulation. Overexpression of CAST blunts the sensitivity to lenalidomide in lenalidomide-sensitive cells, such as MDSL cells. These data suggest that CAST should be restricted for successful execution of GPR68/Ca^2+^/CAPN1-mediated cytotoxicity in MDSL cells.

There are two points that limit the clinical potential of the GPR68/Ca^2+^/CAPN1 pathway in MDS/AML. First, the GPR68 agonist Isx is not an FDA-approved drug. Second, whether the GPR68/Ca^2+^/CAPN1 pro-apoptotic pathway executes its tumoricidal effect in MDS/AML cells is dependent on the expression of CAPN1 and CAST. However, pharmacological compounds that activate CAPN1 or inhibit CAST function are not yet available. Therefore, we have identified alternative pathways that could potentiate the sensitivity to lenalidomide in MDS/AML.

### 6.4. RCAN1

In addition to GPR68, depletion of IRF9, RRP1B or RCAN1 also reverses the inhibitory clonogenicity of MDSL cells in the presence of lenalidomide. RCAN1 is an endogenous inhibitor of the serine/threonine phosphatase calcineurin (CaN, also known as protein phosphatase 3, PPP3) (Figure 5A) [126]. Upon recognizing the antigen peptide/MHC complex on APCs, TCR complex and coreceptor on T cells serve as the “first” signal that activates various downstream signaling pathways, including the CaN/nuclear factor of activated T cells (NFAT) pathway [127]. The co-stimulator receptor CD28 on T cells serves as the “second” signal that fully activates T cell response [66]. These signaling pathways induce proliferation, survival, cytokine production, cytokine receptor expression, differentiation and function of effector T cells [66]. The CaN inhibitor Cyclosporin-A suppresses T cell response and is thus used in clinic to prevent rejection after organ transplantation [64]. The RCAN1-CaN signaling axis is therefore investigated as an alternative candidate pathway to enhance sensitivity to lenalidomide in MDS as well as AML [62].

Similar to GPR68, the levels of RCAN1 transcript and protein are upregulated in MDSL cells after treatment with lenalidomide (Figure 5C) [62]. Knockdown of IKZF1 also upregulates RCAN1 transcripts in MDSL cells, indicating that IKZF1 actions as a transcription repressor for RCAN1 as well [62]. Different from GPR68, the promoter region of RCAN1 gene locus does not contain any binding peaks for IKZF1, indicating alternative mechanisms in IKZF1-mediated transcriptional repression of RCAN1. Ding et al. report that IKZF1 impacts the global regulation of the enhancer/super-enhancer landscape in T cell leukemogenesis [128]. Whether IKZF1 represses RCAN1 transcription through modifying the enhancer/super-enhancer regions on RCAN1 gene locus needs further study. As expected, knockdown of RCAN1 potentiates CaN activation, while treatment with Cyclosporin-A dampens CaN activation in MDSL cells, indicating that MDSL cells exhibit basal level of CaN activation that is inhibited by RCAN1. Treatment with Cyclosporin-A correlates with increased Annexin V^+^ cells and reduced colonies in MDSL cells, indicating that basal level activation of CaN provides a pro-survival signal in MDSL cells that is consistent with its pro-survival function in T cells [127]. After pre-treatment with lenalidomide, co-treatment with lenalidomide and Cyclosporin-A further increases Annexin V^+^ cells and further reduces colonies in MDSL cells than single treatment with lenalidomide, indicating that Cyclosporin-A potentiates the sensitivity of MDSL cells to lenalidomide. Co-treatment with lenalidomide and Cyclosporin-A also correlates with more Annexin V^+^ cells than single treatment with lenalidomide in primary bone marrow cells from MDS patients who are diagnosed with del(5q) MDS or RAEB II. These studies indicate that both lower-risk and higher-risk MDS cells respond more efficiently to lenalidomide in the presence of Cyclosporin-A.

For lenalidomide-sensitive AML cell lines, such as TF-1 cells, co-treatment with lenalidomide and Cyclosporin-A results in more Annexin V^+^ cells than single treatment with lenalidomide. For lenalidomide-resistant AML cell lines, such as HL-60 and THP-1 cells, the addition of Cyclosporin-A results in no alterations on the response to lenalidomide. For lenalidomide-sensitive or -resistant patient-derived xenograft (PDX) models that are derived from relapsed and chemo-resistant AML patients, the addition of Cyclosporin-A significantly increases the anti-leukemia effect of lenalidomide. Of note, these PDX models harbor various genetic mutations and cytogenetic aberrations, including mutations of TP53, TET2, NPM1, FLT3-ITD, PTPN11, MLL arrangements, del(5q), mono7 and complex karyotypes, indicating that Cyclosporin-A enhances the response to lenalidomide in AML irrespective of genetic or cytogenetic alterations. This greatly increases the potential of clinical application of lenalidomide for aggressive types of myeloid malignancies. However, more studies are needed to evaluate the in vivo effect of the combined use of lenalidomide and Cyclosporin-A on MDS/AML.

One concern for the combined use of lenalidomide and Cyclosporin-A for myeloid malignancies is the immunosuppressive effects of Cyclosporin-A on T cells [129]. Cyclosporin-A is an 11 amino acid-cyclic endecapeptide that binds a family of intracellular receptors, the cyclophilins, leading to inhibition of the phosphatase activity of CaN [130]. High doses of Cyclosporine-A restrict dephosphorylation of NFAT, the main downstream substrate of CaN, leading to reduced production of cytokines [131]. Cyclosporin-A selectively suppresses the expression of IL-2 and alpha chain of IL-2 receptor (IL-2Rα), leading to profound reduction of proliferation of CD4^+^ helper T cells and CD8^+^ cytotoxic T cells during an immune response (Figure 5A) [132,133,134]. High-dose Cyclosporin-A (>20 mg/kg per day) is used as an immunosuppressive drug to alleviate rejection after organ transplantation [135].

In addition to immunosuppressive effects, growing evidence indicates immunomodulatory effects of Cyclosporin-A, especially at low doses [129]. Cyclosporin-A impacts the induction of delayed-type hypersensitivity, reprogramming an antibody response to a cell-mediated immune response [136]. Cyclosporine-A is also involved in several autoimmune diseases, such as arthritis, thyroiditis, uveitis, myocarditis and experimental autoimmune encephalomyelitis (EAE) in humans and other animals [137]. High-dose Cyclosporine-A abrogates EAE and autoimmune response in Lewis rats, while low-dose Cyclosporine-A induces a relapse that is accompanied by increased production of IFN-γ [138]. Low-dose Cyclosporine-A inhibits the immunosuppressive activity of human CD4^+^CD25^+^ Tregs in vitro, which is associated with increased levels of IL-2 and IFN-γ in cell culture [139]. In contrast, high-dose Cyclosporine-A results in no alteration on the activity of Tregs [139]. In a skin transplant mouse model, control mice exhibit allograft rejection within 2 weeks with infiltration of phagocytes in skin and CD8^+^ T cells in spleens and increased levels of cytokines in serum, including IL-2, IFN-γ and TNF-α [140]. Mice treated with therapeutic doses of Cyclosporin-A (15 mg/kg) exhibit no rejection, which is associated with reduced infiltration of CD8^+^ T cells in spleens and lower levels of IL-2, IFN-γ and TNF-α in serum compared to control mice. Mice treated with minimal doses of Cyclosporine-A (5 × 10^-55^ mg/kg) exhibit spontaneous premature allograft rejection within 1 week, which is associated with higher levels of IL-2, IFN-γ and TNF-α in serum compared to control mice. Intriguingly, the amount of CD4^+^CD25^high^FoxP3^+^ Tregs is largely diminished in mice with low-dose group than control and high-dose groups, indicating that low-dose CsA also blunts the function of Tregs in vivo.

Cyclosporine-A, especially at low doses, is used for other pathological disorders, such as severe psoriasis, uveitis and urticaria [141,142,143]. Cyclosporine-A is also being tested for patients with solid tumors or hematologic malignancies as monotherapy or in combination with other drugs. For example, advanced non-small cell lung carcinoma patients treated with low-dose Cyclosporine-A (1–2 mg/kg) in combination with etoposide/cisplatinum (EP) exhibit longer survival time than those treated with high-dose Cyclosporine-A (3–6 mg/kg) and EP [144]. Patients with peripheral T cell lymphoma or cutaneous T cell lymphoma are treated with Cyclosporine-A (7.5 mg/kg), but most patients do not respond to Cyclosporine-A [145]. A patient with advanced thymoma has remained in long-term complete remission by using low-dose Cyclosporine-A [146]. Patients with lower-risk MDS are treated with methylprednisolone with or without Cyclosporine-A, and one third of the patients show a hematologic response [147]. MDS patients, mostly with refractory anemia, are treated with Cyclosporine-A (4 mg/kg), and almost half of the patients (8 out of 19 patients) show an erythroid response [148]. In a different study, MDS patients are treated with Cyclosporine-A, and over half of the patients (7 out of 12 patients) show erythroid response [149]. Cyclosporine-A is evaluated in patients with MDS or severe aplastic anemia in combination with anti-thymocyte globulin (ATG) and exerts superior response compared to other immunosuppressants [150,151,152].

To understand the combined effect of lenalidomide and Cyclosporin-A on T cell response, mouse T cells are activated by engaging the “first” signal (i.e., anti-CD3 mAb) and the “second” signal (i.e., anti-CD28 mAb), followed by measurement of T cell proliferation [62]. Single treatment with lenalidomide does not change T cell proliferation, while co-treatment with lenalidomide and Cyclosporine-A inhibits T cell proliferation, indicating that Cyclosporin-A inhibits T cell response. Intriguingly, pre-treatment with lenalidomide, followed by co-treatment with lenalidomide and Cyclosporin-A, does not inhibit T cell proliferation, indicating that pre-treatment of lenalidomide primes T cells, which reverses the inhibitory effect of Cyclosporin-A on T cell response. Given that lenalidomide does not act through mouse Crbn [59], our studies indicate that lenalidomide primes mouse T cells that is independent on the function of the CRL4^CBRN^ complex. Following CD3 ligation or in response to APCs, lenalidomide acts as a co-stimulator through triggering phosphorylation of CD28, leading to T cell activation (Figure 5A) [67]. This might explain how lenalidomide reverses the immunosuppressive action of Cyclosporin-A in mouse T cells. Given that Crbn is not involved in this process, it is serving as a mechanism of lenalidomide-mediated immunomodulation that is independent on the function of the CRL4^CBRN^ complex (i.e., CRL4^CRBN^-independent).

## 7. Conclusions

Despite the superior hematologic and cytogenetic response to lenalidomide in lower-risk del(5q) MDS, clinical efficacy of lenalidomide in higher-risk MDS and AML without del(5q) or with other cytogenetic aberrations remain limited [153]. Mechanistic studies have uncovered novel signaling pathways that confers therapeutic vulnerability to lenalidomide in combination with other drugs, especially FDA-approved ones. For example, we have found that Cyclosporin-A enhances the sensitivity to lenalidomide in lower-risk and higher-risk MDS as well as AML, irrespective of genetic or cytogenetic abnormalities. Of note, we also find that pre-treatment with lenalidomide reverses the immunosuppressive effect of Cyclosporin-A through a CRL4^CBRN^-independent manner. Our studies provide therapeutic rationale of combined use of lenalidomide and Cyclosporin-A for the treatment of myeloid malignancies. However, in vivo assays in animal models are urgently needed before any translational studies. Our discoveries also raise an open question: can we use immunomodulatory drugs and immunosuppressive drugs together for the treatment of myeloid malignancies? To answer this question, more work is needed from hematologists as well as immunologists.
